# The Anti-Inflammatory Properties of Phytochemicals and Their Effects on Epigenetic Mechanisms Involved in TLR4/NF-κB-Mediated Inflammation

**DOI:** 10.3389/fimmu.2021.606069

**Published:** 2021-03-26

**Authors:** Haidy A. Saleh, Mohamed H. Yousef, Anwar Abdelnaser

**Affiliations:** ^1^Department of Chemistry, School of Sciences and Engineering, The American University in Cairo, Cairo, Egypt; ^2^Department of Pharmacology and Toxicology, Faculty of Pharmacy, The British University in Egypt, Cairo, Egypt; ^3^Biotechnology Graduate Program, School of Sciences and Engineering, The American University in Cairo, Cairo, Egypt; ^4^Institute of Global Public Health, School of Sciences and Engineering, The American University in Cairo, Cairo, Egypt

**Keywords:** inflammation, flavonoids, miRNAs, TLR4 signaling pathway, innate immunity

## Abstract

Innate immune response induces positive inflammatory transducers and regulators in order to attack pathogens, while simultaneously negative signaling regulators are transcribed to maintain innate immune homeostasis and to avoid persistent inflammatory immune responses. The gene expression of many of these regulators is controlled by different epigenetic modifications. The remarkable impact of epigenetic changes in inducing or suppressing inflammatory signaling is being increasingly recognized. Several studies have highlighted the interplay of histone modification, DNA methylation, and post-transcriptional miRNA-mediated modifications in inflammatory diseases, and inflammation-mediated tumorigenesis. Targeting these epigenetic alterations affords the opportunity of attenuating different inflammatory dysregulations. In this regard, many studies have identified the significant anti-inflammatory properties of distinct naturally-derived phytochemicals, and revealed their regulatory capacity. In the current review, we demonstrate the signaling cascade during the immune response and the epigenetic modifications that take place during inflammation. Moreover, we also provide an updated overview of phytochemicals that target these mechanisms in macrophages and other experimental models, and go on to illustrate the effects of these phytochemicals in regulating epigenetic mechanisms and attenuating aberrant inflammation.

## Introduction

The innate immune system is the non-specific, inherited immune defense mechanism encoded in the germ-line genes of the host (1). It initiates a rapid response and recruits immune cells promptly to the site of infection or inflammation through cytokines and chemokines production (2, 3). The most important cellular components of the innate immune system are neutrophils and macrophages, which are effective phagocytes that act as the first line of defense against foreign bodies (3, 4). These cells are known as antigen-presenting cells (APCs) that usually recognize pathogens through their surface-expressed receptors, known as pattern recognition receptors (PRRs), which bind to molecular patterns expressed on the surfaces of invading microbes (5). Upon engulfment of a pathogen, antigen presenting macrophages express antigen peptides derived from the engulfed pathogen on the immune cell surface *via* the major histocompatibility complex (MHC) class II, in order to recruit CD4^+^ T cells, one type of adaptive immune cells (6, 7). This connection between phagocytic immune cells and T-lymphocytes, therefore, shapes the link between innate and adaptive immunity.

The innate immune response begins when pattern recognition receptors (PRRs) expressed on immune cells detect either pathogen-associated molecular patterns (PAMPs), such as microbial nucleic acids, lipoproteins, and carbohydrates, or damage-associated molecular patterns (DAMPs) released from damaged cells (5, 8). Then oligomerization of the receptor ensues, followed by the assembly of the activated PRRs subunits, which initiates signaling cascades leading to the activation of mediators that attract leukocytes to the site of infection or injury (5, 8). Subsequently, these leukocytes, including macrophages, neutrophils, and dendritic cells phagocytose microbial elements and release more proinflammatory cytokines, such as TNF-α, IL-6, IL-12, and type I and II interferons (IFNs), which collaboratively attempt to contain the pathogen until highly specific, activated cells of the adaptive immune response are recruited to completely eliminate the infection (6, 8).

A hallmark of the innate immune response is inflammation, which is a complex set of defense mechanisms acting in concert to restore homeostasis in body systems after injuries or infections (8, 9). Inflammatory reactions are mainly a result of the vasodilation due to the release of histamine, prostaglandins (PGs), and nitric oxide (NO) that leads to a noticeable increase in blood flow and accumulation of circulating leukocytes (8). Additionally, proinflammatory cytokines secreted from activated immune cells, such as tumor necrosis factor-alpha (TNF-α), interleukin-1 (IL-1) and interleukin-6 (IL-6) enhance the vascular permeability of leukocytes through raising the levels of leukocyte adhesion molecules on endothelial cells (5, 10). Usually, inflammation is triggered to restore homeostasis and repair tissues; however, prolonged inflammation could lead to serious problems, including cellular dysregulation as observed with cell senescence, impaired proteolysis and apoptosis, and further tissue dysfunction (11). Recently, many studies correlated inflammatory disturbance with epigenetic modifications (11).

Epigenetic mechanisms modulate differential gene expression with no alteration to the DNA sequences (12). Epigenetic processes include modifications to DNA, such as DNA methylation or histone proteins, including histone methylation, acetylation and acylation, and they also involve microRNAs (13). Epigenetic modifications are potentially reversible, and, therefore, an in-depth understanding of these changes may help identifying new therapeutic targets (14). A plethora of reviews covered distinct pathways of innate immunity, inflammation and highlighted the relationship between TLR4 signaling and inflammatory diseases and cancer (15, 16). The current review focuses on the various regulatory epigenetic mechanisms involved in inflammation and summarizes the recent findings of targeted phytotherapy.

## TLR4 Signaling in Innate Immune Response

One of the essential and first-identified members of PRRs are TLRs (5, 17). They are expressed on various immune cells, including macrophages, dendritic cells (DCs), B cells, specific types of T cells, and even on non-immune cells such as cardiac cells, fibroblasts and epithelial cells (1). TLRs are type I transmembrane proteins characterized by the extracellular leucine-rich repeats (LRRs) that recognize different microbial epitopes and has a cytoplasmic signaling domain similar to that of the interleukin 1 receptor (IL-1R), called Toll/Interleukin 1 receptor (TIR) domain, which is responsible for signal transduction (1, 4, 5, 18). The TLR family was first identified in Drosophila flies, and now twelve members of the TLR family have been identified in mammals (5, 18). Grouped as subfamilies, TLR1, TLR2, and TLR6 recognize lipids, while TLR7, TLR8, and TLR9 recognize nucleic acids (6, 8). Some receptor/ligand pairs are commonly known, such as TLR4 and LPS, TLR5 and flagellin, TLR 1, 2, and 6 with lipoproteins (18). In addition, TLRs are either expressed on the cell-surface, e.g. TLRs 1, 2, 4, 5, and 6, or internalized to the endosome, e.g. TLRs 3, 7, 8, and 9 (5, 6, 18). Together with phagocytic-antigen presentation, the activation of TLRs leads to the expression of inflammatory cytokines, which further recruits antigen-specific cells (18).

When activated, TLRs, in turn, activate various genes that function to moderate host defense, including inflammatory cytokines, chemokines, MHC and co-stimulatory molecules (18). Mammalian TLRs also induce multiple effector molecules, such as iNOS and antimicrobial peptides that can directly destroy microbial pathogens (8). Depending on TIR domains, TLRs activate NF-κB and MAPK (mitogen activated protein kinases) and induce target genes (5, 18). Upon ligand binding (e.g. LPS), TLR4 dimerizes and induces the recruitment of intracellular adaptor proteins that trigger two standard models of signaling cascades: myeloid differentiation primary response gene 88 (MyD88)-dependent and Toll-interleukin-1 receptor domain-containing adaptor inducing interferon-beta (TRIF)-dependent pathways (8, 18).

**Figure 1:**
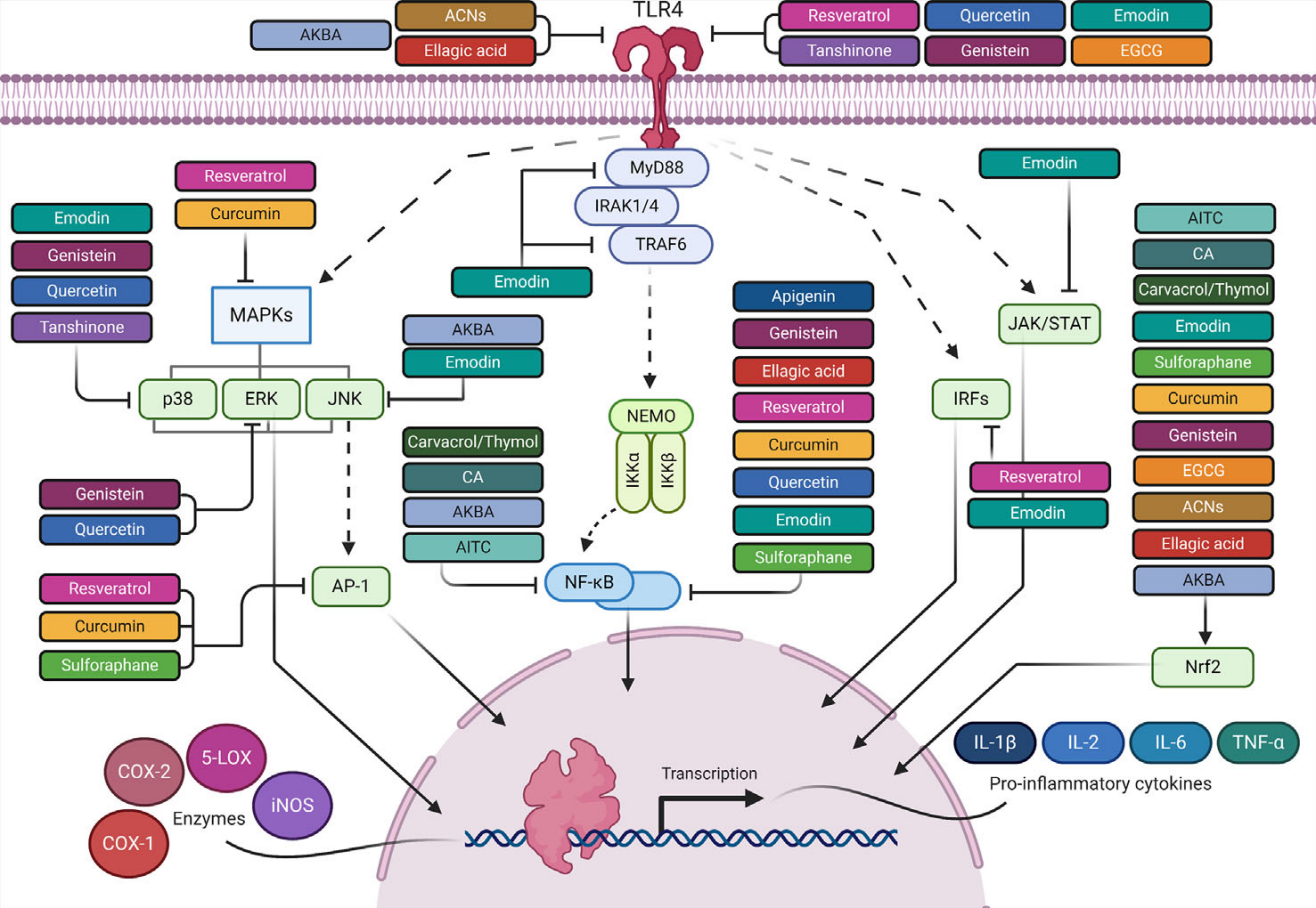
The anti-inflammatory mediated effects of phytochemicals along the TLR4 signaling pathway. Created with Biorender.

**Figure 2:**
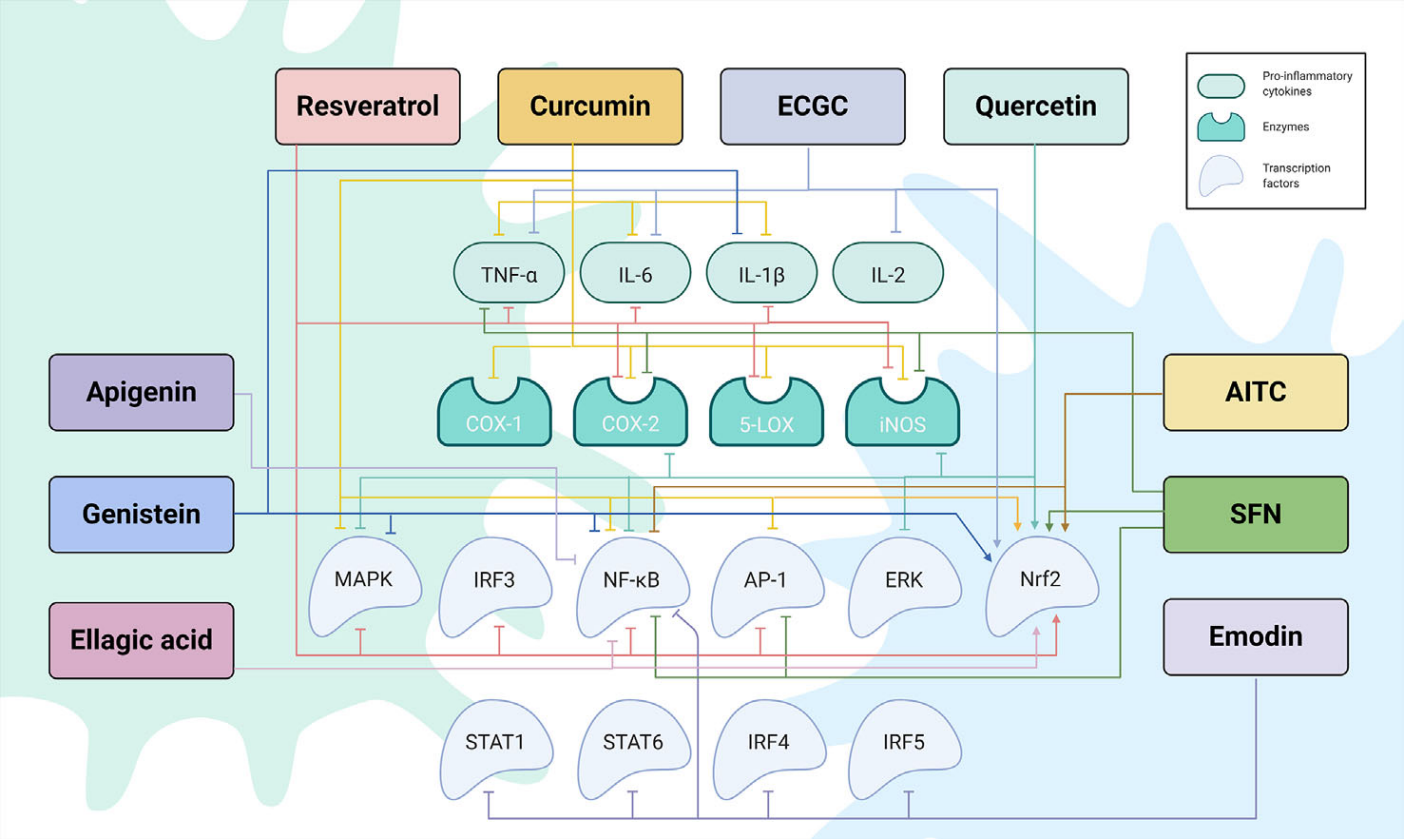
The regulatory network by which phytochemicals affect TLR4-mediated inflammatory responses. Created with Biorender.

The *MyD88-dependent pathway* originates from the cytoplasmic TIR domain (19). The activation of MyD88 causes the autophosphorylation of interleukin-1 receptor-associated kinases (IRAK), namely IRAK1, IRAK2, and IRAK4, which associate temporarily with TNF receptor-associated factor 6 (TRAF6) (8, 18). This autophosphorylation and oligomerization for IRAK and TRAF6, respectively, finally leads to the activation of IκB kinase (IKK) (in response to TAK1/TAB complex activation) and mitogen-activated protein kinase (MAPK), namely ERK, JNK, p38 (10, 18). Then, ensuing signal dissemination results in the activation and the translocation of nuclear factor kappa B (NF-κB) to the nucleus and the subsequent activation of the activator protein-1 (AP-1) transcriptional program (8). Both NF-κB and AP-1 control inflammatory responses through the induction of inflammatory cytokines, such as TNF-α, IL-12, and others (8, 18). The *TRIF-dependent pathway*, which mediates the late phase activation of NF-κB, primarily recruits TRIF and this results in the ubiquitination of TNF receptor-associated factor 3 (TRAF3) which induces TANK-binding kinase 1 (TBK1) binding to IκB (inhibitor of NF-κB) kinase epsilon (IKKϵ)) (8, 20–24). Thenceforth, the TBK1-IKKϵ complex phosphorylates the transcription factor interferon regulatory factor 3 (IRF3), ultimately driving the expression of interferon-beta (IFN-β), which induces STAT1-dependent genes encoding monocyte chemoattractant protein 5 (MCP-5), IFN-inducible protein 10 (IP-10) and iNOS (25, 26) (**Figures 1** and **2**).

Despite the great progress in understanding the molecular mechanisms underlying inflammation-related signaling events of TLRs, particularly TLR4, less data is available about the role of epigenetics in regulating the transcriptional responses downstream of the TLR system (27). Recent studies have demonstrated that TLR-induced epigenetic alterations forms a solid platform in both positive and negative regulation of activated inflammatory genes (27).

## Role of Epigenetic Regulation in Inflammation

A balanced interplay of the chromatin machinery is pivotal in regulating the transient induction of TLR-related genes (28). Changes in chromatin play an essential role in both physiological and pathological processes through responding to environmental signals and regulating gene transcription (29). Epigenetic regulations mainly include DNA modifications, histone post-translational modifications (PTMs), chromatin remodeling and microRNAs (miRNAs), which together function in a coordinated manner (30). These epigenetic modifications occur with the aid of several catalytic enzymes that add or remove chemical moieties, including DNA methyltransferases (DNMTs), histone methyltransferases (HMTs), histone demethylases (HDMs), histone acetyltransferases (HATs), and histone deacetylases (HDACs) (13).

### Histone Modification

Histone modifications, such as acetylation and methylation, are critical regulators of gene transcription (31). These modifications can regulate the binding of effector molecules to DNA and, therefore, control transcription, repair, and replication processes (31). Acetylation of histones is associated with an “open” chromatin conformation that activates transcription (14). *Histone acetylation* of the lysine residues is regulated by histone acetyltransferases (HATs), which add an acetyl group and mediate histone hyperacetylation towards euchromatin and gene upregulation, whereas histone deacetyl transferases (HDATs), which remove the acetyl group, induce histone hypoacetylation towards heterochromatin and silencing of inflammatory genes (14, 32). Villagra et al. showed that promotors of several proinflammatory cytokines are acetylated and they, usually, display reduced HDAC activity, leading to transcriptional activation (33). Not only this, but also histone H3 acetylation (H3Ac) at the promoters of many cytokines and chemokines increases NF-κB recruitment to these regions, following inflammation (34). In this context, Hargreaves and colleagues stated that H3Ac modification primarily occurs at promotors of primary response genes (PRG), which are rapidly induced by TLR4 following macrophage activation (35). Furthermore, Wu and Chiang showed that upon TLR ligation, an inducible addition of histone H4Ac at lysine 5, 8, and 12 (H4K5, H4K8, and H4K12 Ac) at PRG promoters takes place, facilitating the binding of an epigenetic reader, called BRD4 that recognizes inducible acetylation marks on histones and recruits positive transcription factors for active transcription (36, 37). These chromatin modifiers are employed in regulating innate signaling pathways induced by infection, and regulate the expression of PRR signal transducers and inflammatory genes through initiating various histone modifications (29). TLR4 is one of the most important PRR signal transducers. It has been implicated in the epigenetic regulation of the TNF-α promoter, where LPS stimulation increased the H3 and H4 acetylation of the TNF-α locus, and thus induced gene transcription (38).

On the other side, *Histone deacetylases (HDACs)* reverse the activity of HAT by removing acetyl groups from histones, making chromatin more condensed and thus, promoting gene inactivation (39). It has been reported that HDAC recruitment is associated with the pathogenesis of diseases, including inflammation and cancer, and is targeted by therapeutic agents in patients (40). Inhibitors of HDACs have pleiotropic effects on the immune response, especially on TLR signaling; they are known to suppress different cytokines, such as IL-6, IL-10, TNF-α, IL-12, and IL-23 (41). Moreover, inhibitors of class I, II, and IV HDACs are found to inhibit LPS-induced upregulation of TLRs, CD14, and MD-2 genes (40). In this respect, reduced expression and activity of HDAC2, in particular, was noticed with macrophage tolerance, which was reported in several inflammatory models, such as lung macrophages, biopsies, and blood cells from patients with COPD, severe asthma, and smoking-induced asthma (42).

In contrast to histone acetylation that occurs primarily on lysine residues, *histone methylation* takes place on lysine, arginine and other amino acid residues in histone protein tails, and eventually modifies chromatin in either its activated or repressed states (43). For example, tri-methylation of histone H3 on lysine 4 and 36 (H3K4me3 and H3K36me3) are commonly referred to as activators of transcription (44). Nonetheless, histone methylation on lysines 9 and 27 (H3K9me3 and H3K27me3) facilitates gene silencing (45). Histone methyltransferases (HMT) are histone-modifying enzymes that regulate gene expression by catalyzing the transfer of methyl groups to lysine and arginine residues of histones (H3 and H4) at specific sites (46). The number of methyl groups transferred and the histone residues involved determine whether gene transcription is suppressed or activated (47). Multiple reports demonstrated the key role of H3K4me3 in positively regulating the TLR4 signaling pathway in macrophages during the LPS response. For example, increased levels of H3K4me3 at the gene promotor induced the transcription of PIGP, a product required for proper membrane anchoring of CD14 in primary macrophages for TLR4-mediated signaling (48). Another report showed that elevated levels of H3K4me3 at *Socs1* promoter resulted in increased expression of suppressor of cytokine signaling 1 (SOCS1), which acts as a negative regulator of TLR4-induced inflammation (49). Similarly, increased H3K4me3 levels on *Tnfaip3* promotor upon LPS stimulation in macrophages results in transcribing the *Tnfaip3-*encoded ubiquitin-editing enzyme A20, which in turn mediates K63 deubiquitylation of TRAF6 and NF-κB essential modulator (NEMO), therefore suppressing TLR4-mediated signal transduction (50). Hargreaves and colleagues conducted a ChIP analysis of RNA pol II-associated genes that shows the elevated basal levels of H3K4me3 modification in the promotors of many primary response genes (PRGs) to be usually characteristic of active transcription (35). On the contrary, the secondary response genes (SRGs), which have delayed induction, showed remarkable H3K4me3 modification only after TLR ligation, and not at the basal level like PRGs (35).

On the contrary, other histone methylations, such as H3K27me3 possess a clear suppressive effect on gene transcription. For instance, induced H3K27me3 levels at the promoter region of Tollip gene (a negative regulator of TLR pathway) inhibited Tollip transcription and, therefore, activation of the TLR signaling cascade (51). Moreover, the relationship between epigenome reprogramming and inflammation might also be expressed in macrophage polarization. Following NF-κB activation, macrophages induce a protein called Jmjd3 that binds to PcG target genes (a Polycomb Group proteins, which are involved in development and differentiation of macrophages) and regulates their H3K27me3 levels and, thus their transcriptional activity (52). Taken together, these interesting differences show the significant influence of chromatin epigenetic modifiers on different immune responses and time-dependent production of TLR-responsive genes.

### DNA Methylation

DNA methylation involves a covalent attachment of a methyl group to the cytosine residue at cytosine-phosphate-guanine (CpG) site by DNA methyltransferase enzymes (DNMTs) (47). These CpG sites are present in about 70% of human gene promoters, and are essential modulators of gene transcription (53). Usually, DNA hyper-methylation induces chromatin condensation (heterochromatin) and enhance gene repression, inhibiting the binding of transcription factors at promoter sites on genomic DNA (54, 55). DNA methylation by DNMTs also induces coupling with other gene repressing proteins such as HDACs (56). Indeed, DNA methylation is highly imperative in regulating inflammatory genes. For example, epigenome-wide association studies (EWAS) linked DNA hypomethylation with increased inflammation (57, 58). In addition, DNA hypomethylation with aging has been suggested to be the cause of chronic inflammation and cancer (59). While, another study by Hahn et al. reported that atypical DNA methylation of some Polycomb group (PcG) protein targets in mammalian genome results in their efficient downregulation after chronic inflammation (60). Cooperatively, modifications in both DNA methylation and histone acetylation of genome status can regulate TLR4 signaling (61). Of interest, DNMT3A, the highly expressed DNA methyltransferase in macrophages, has been shown to indirectly induce the expression of type I IFNs during virus infection *via* maintaining increased HDAC9 expression, which, in turn, maintained the deacetylation status of the key TLR4 signaling molecule TBK1 and, thus, enhanced its kinase activity (29, 62).

As DNA methylation is a common epigenetic modification in inflammation-related diseases, it would be interesting to examine the effects of individual natural products on DNA methylation and inflammation-associated cascades in humans (27). Notably, the post-transcriptional modifications of inflammatory genes is suggested to be mediated by two distinct regulation levels of TLR4 signaling pathway (63). The first level is mediated by epigenetic alterations, and the second level is regulated by the differential expression of TLR4-responsive miRNAs and, specifically, miR-146a, miR-155, and miR-21 (14).

### MicroRNAs

**Figure 3:**
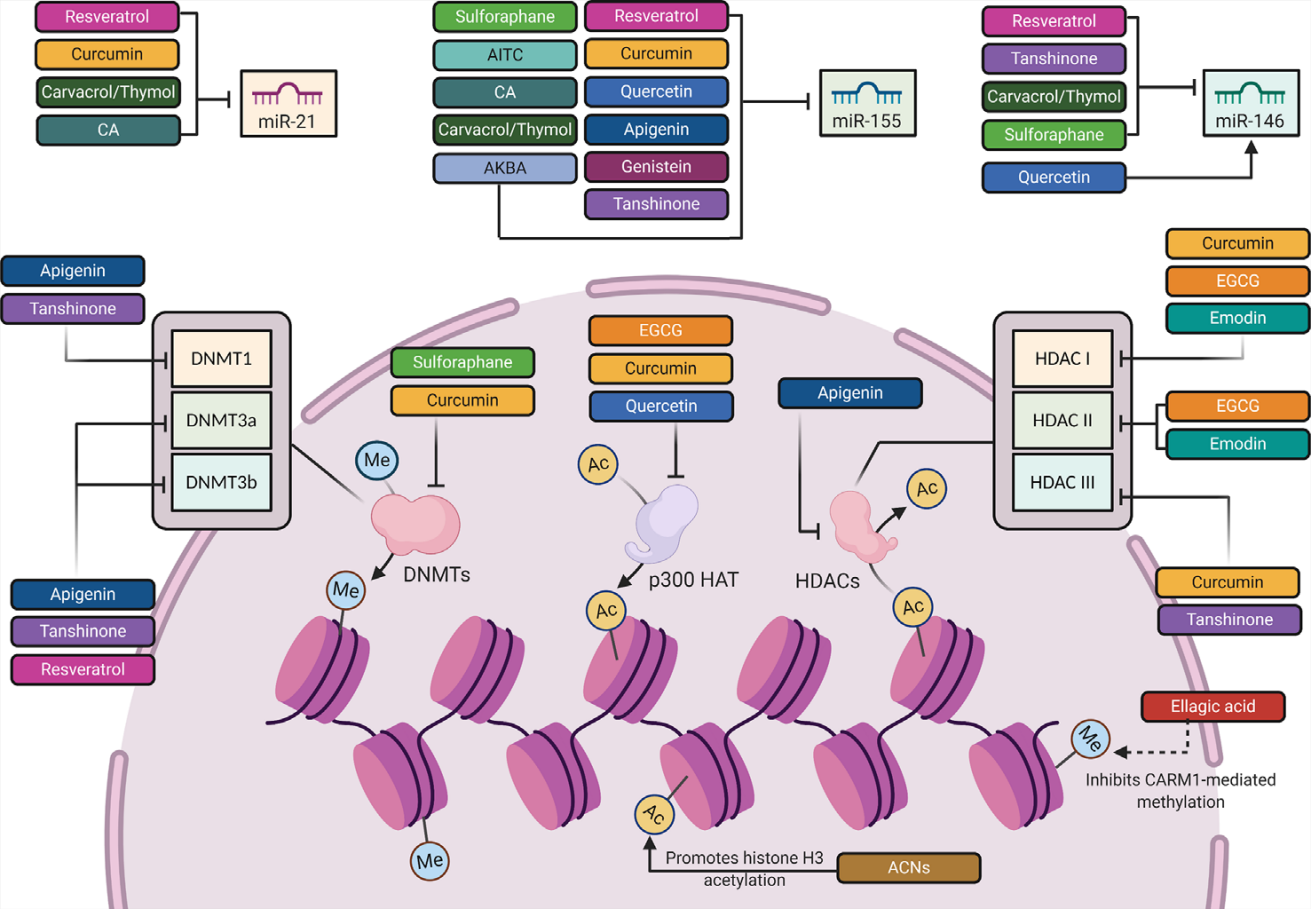
The epigenetic mechanisms involved in the regulation of TLR4 signaling pathway by phytochemicals. Created with Biorender.

Recently, a plethora of research studies have focused on deciphering the role of miRNAs in the regulation of inflammatory gene expression (64). MiRNAs are short double-stranded, non-coding RNA molecules that are ~22 nucleotides in length (65). They bind to the 3` untranslated region (UTR) of a target mRNA sequence causing gene down-regulation at the post-transcriptional level, and inducing translational arrest (66). miRNAs are currently viewed as essential regulators in key immune responses, for example regulation of maturation, proliferation, differentiation and activation of both innate and adaptive immune cells (67). TLR-responsive miRNAs are either up-regulated or downregulated after LPS treatment (67). Although they are heritable, miRNAs are also inducible and reversible (68). This flexible nature of miRNA expression is obvious in inflammatory reactions, which are primarily dependent on the surrounding environment. For instance, the expression of most miRNAs is triggered in an NF-κB-dependent manner after TLR stimulus (65). The expression of miRNAs is dependent on TLR stimulation, highlighting the cause/effect relationship between LPS stimulation and the upregulation of miR-146a and miR-155 in human monocytes (69–73). In terms of response to TLR4 pathway activation, miRNAs are classified as either “early response miRNAs”, expressed rapidly after LPS stimulation such as miR-146 and miR-155 or “late response miRNAs”, expressed in macrophages at a later time after LPS treatment such as miR-21 (69–73). miR-146a-5p, miR-155-5p, and miR-21 are also involved in the regulation of TLR downstream signaling through TLR-induced transcriptional factors (74, 75). They target cytokines such as type I IFNs, TNF-α, IL-6, IL-12, and IL-10, and this has been indicated by the presence of binding sites for miRNAs on the mRNAs encoding these cytokines and chemokines (76–78). The miRNAs, miR-21, miR-146a, and miR-155 are particularly predominant in the majority of inflammation-related studies, because of their expression succeeding TLR stimulation, especially in macrophages (67) (**Figure 3**).

#### miR-146a

miR-146a is a pivotal repressor of NF-κB inflammatory signaling in several cell types. It is one of the miR-146 family that is present in chromosome 5 and 10 (67, 79–81). Upregulation of miR-146a has been reported in inflammatory diseases, such as osteoarthritis and rheumatoid arthritis (82). A recent study illustrated that miR-146a negatively regulated TLR4 signaling through blocking TRAF6 and IRAK1, which activates downstream transcription factors NF-κB and AP-1 (14, 71, 83–86). It has been reported that increased expression of TRAF6 was observed in 5q chromosomal deficient models, leading to impaired innate immune signaling and causing leukemia and bone marrow failure (87). In addition, IRAK2 and IRAK4 have been recognized as targets of miR-146a, which results in decreasing inflammatory cytokines (71, 83, 88). Another study illustrated the role of miR-146a as a negative regulator of type1 IFN response in human peripheral blood mononuclear cells (PBMCs) (89). Moreover, in LPS-treated human monocytes, miR-146a has been shown to degrade mRNA transcripts of IRF3, a transcriptional factor responsible for type1 IFN production (90). miR-146a has also been reported to be involved in regulating cytokines release and apoptosis in human dendritic cells (71, 91). From this angle, a recent study showed that by targeting IRAK1, IRAK2, and TRAF6 in LPS-stimulated macrophages, miR-146a sequentially suppresses the production of type I IFNs, and the cytokines TNF-α, IL-1β, and IL6 (83, 85)

#### miR-21

Second, miR-21 is a cancer-associated miRNA that is induced by NF-κB activation, and acts as a negative regulator of TLR signaling inflammatory responses (67, 92). It has been revealed that miR-21 inhibits MyD88 and IRAK1 expression during hepatitis C viral infection in PBMCs (93). Furthermore, Sheedy et al. highlighted the role of miR-21 in downregulating the expression of programmed cell death protein 4 expression (PDCD4) in LPS-induced RAW 264.7 macrophages (94). PDCD4 acts as a tumor suppressor protein that activates the proinflammatory mediators NF-κB and IL-6, and suppresses the anti-inflammatory cytokine IL-10, which inhibits of the microRNA, miR-155 (94, 95). MiR-21, therefore, has anti-inflammatory effects by increasing the production of IL-10 and suppressing NF-κB activity (67, 94).

#### miR-155

Another tumor-associated miRNA is miR-155. miR-155 has a significant role in the TLR-mediated immune response and can target related signaling proteins of the NF-κB pathway (65). A positive correlation exists between miR-155 overexpression and NF-κB activation as evidenced by Baltimore et al. in their study, which linked miR-155 upregulation with mammalian inflammatory reactions (71, 96). Another study by Tili et al. showed that miR-155 expression can have both positive and negative effects on NF-κB signaling proteins however, they supported a positive regulatory role of miR-155 in NF-κB pathway, which was evidenced by increased serum TNF-α in miR-155 transgenic mice (97).

As a positive regulator of TLR4 signaling pathway, miR-155 suppresses two negative regulators of TLR4-induced inflammation, SOCS1 and SH2 (Src homology 2)-containing inositol phosphatise-1 (SHIP-1) (98, 99). This suppression of these two important negative regulators of TLR4 signaling results in boosting MAPK activity and stimulating the expression of inflammatory cytokines in primary macrophages and dendritic cells isolated from mice (67, 100, 101). On the other hand, Ceppi et al. showed that, sometimes, miR-155 exerts an anti-inflammatory effect through targeting TAB2, thereby inhibiting TAK1-dependent stimulation, and further NF-κB and MAPK activation in human monocyte-derived DCs (102). On account of the above findings, miR-155 remains an interesting and significant player in downstream inflammatory pathways (67).

As demonstrated hereinabove, the significance of miRNAs in inflammatory processes has directed research towards a better understanding of their functions and interactions in TLR4 signaling. That being said, plant-derived bioactive compounds have ushered in a fresher phase of research in this area. These naturally-occurring agents have been proven to be a promising therapeutic recourse that could interfere, at the genomic level, with TLR4 signaling and modulating miRNA upregulation after NF-κB activation in multiple inflammatory reactions. In this regard, a rundown of bioactive phytochemicals and their proposed mechanism(s) of action within the TLR4 pathway are included in this review, highlighting their potential for post-transcriptional and epigenetic modulation. In brief, we considered three main epigenetic processes of gene repression: DNA methylation, histone modifications, and microRNAs targeting. However, alterations in histone modifications, DNA methylation, and miRNA regulation still need future investigations to provide a better understanding of the molecular basis for various chronic inflammatory diseases. It has to be highlighted that understanding the role of epigenetic modifications is becoming fundamental in human diseases, and since these epigenetic alterations can regulate several inflammatory signaling cascades, they indeed do play an integral part in inflammation (103–106). Therefore, knowing more about epigenetic events during inflammatory responses, and how epigenetic regulation is mediated by TLR signaling during inflammation is worth further exploration, since it opens up opportunities for developing therapeutic interventions. For example, histone deacetylase inhibitors and demethylating agents are currently being proposed for epigenetic therapy (14).

## Plant-Derived Compounds Modulating the TLR4/NF-κB Pathway and Their Associated Epigenetic Regulations

Originally known as secondary metabolites, phytochemicals are plant-synthesized compounds possessing health effects (107). Plant-derived bioactive compounds can be classified as phenolic compounds, including flavonoids and tannins, glucosinolates, alkaloids, and terpenoids (108). When consumed by humans, they get involved in different biological processes inside the body, such as redox processes, cell signaling and, inflammation (109–112). Nowadays, natural products are becoming a promising source for the treatment of several inflammatory conditions (113, 114). Interestingly, the most promising anti-inflammatory herbal extracts were identified to influence key TLR4 signaling pathways and macrophage repolarization (115). Evidence suggests that phytochemicals can attenuate the expression of proinflammatory genes, and promote anti-inflammatory genes; this differential gene expression is regulated by epigenetic modifications (116, 117). For instance, epigenome-wide association studies (EWAS) have demonstrated that differential methylation of inflammatory genes in peripheral white blood cells was associated with diets rich in phytochemicals (27, 118, 119).

In addition, many plant-derived compounds were recently suggested to exert their anti-inflammatory effect through regulating the expression of proinflammatory miRNAs, especially those upregulated after NF-κB activation (120). Although a few reports indicate the precise mechanisms that regulate or deregulate the expression of miRNAs, growing evidence suggests that phytochemicals regulate the expression of miRNAs by interfering with the processes associated with the miRNAs processing and maturation, and these phytochemical-mediated alterations in miRNAs biosynthesis machinery could in part contribute to the miRNAs dysregulation by either increasing or decreasing their levels (121, 122).

In this section, we will discuss, in detail, how some natural compounds can epigenetically alter inflammatory genes, recapitulating on current studies that link phytochemicals-mediated epigenetic modifications to inflammation-associated diseases.

### Polyphenols

Phenolic compounds are involved in numerous signaling pathways, and most importantly, in regulating the redox system and modulating the immune response through inhibiting inflammatory cytokines synthesis (123). Unfortunately, polyphenols, in general, are limited by their pharmacokinetics, such as their poor bioavailability and rapid metabolism (124–131).

#### Resveratrol

A natural phenolic stilbene derivative, resveratrol (RES, 3, 4′,5-trihydroxystilbene) is a phytoalexin that acts as a plant defense mechanism against infection. It is found in grape skins, berries and peanuts (124, 132). This phenolic compound exists in both trans- and cis- isoforms, but the trans-isomer is more stable (132). It is usually taken at a daily dose of 50 to 500 mg (132). Fortunately, no significant adverse effects have been reported for RES, except for an antiplatelet activity that should be monitored, especially if taken with another prescribed antiplatelet or anticoagulants (132–139). Over 40 clinical trials were published in PubMed on the applications of RES in inflammatory disorders, including diabetes, obesity, and coronary artery disease (124). Pharmacologically, RES has been widely recognized for its remarkable anti-mutation, anti-inflammatory, and antioxidant activities (124, 132). By virtue of its anti-inflammatory effects, RES also has neuroprotective, cardioprotective and chemotherapeutic properties (131, 132, 140, 141).

##### RES Mechanisms of Action as an Anti-Inflammatory Agent

Numerous studies have been conducted to provide in-depth insights into the powerful antioxidant and anti-inflammatory function of RES. In a dose-effect relationship, RES exerts its effects at multiple levels (**Table 1**, **Figures 1** and **2**). It inhibits TLR4 and MyD88 expression in activated RAW 264.7 macrophages (142, 143) (**Table 1** and **Figure 1**). Going downstream, RES inhibits NF-κB, MAPK, IRF-3 and AP-1 transcription factors, as well as, iNOS, COX-2, and 5-LOX enzymes (124, 143–147) (**Table 1** and **Figures 1** and **2**). This, in turn, reduces NF-κB-induced proinflammatory cytokines, including TNF-α, IL-6, and IL-1β, and the free radicals, NO and ROS, and LTs and PGs levels (124, 132, 140, 142, 147, 148, 301) (**Table 1** and **Figure 2**). These results were confirmed in LPS-stimulated RAW 264.7 cells, and macrophages isolated from C57BL/6 and BALB/c mice (302). In addition to studies performed on macrophages, others were conducted on heart tissues of rats to investigate the anti-inflammatory effects of RES in response to TLR4/NF-κB-mediated cardiac inflammation (303). These studies showed the cardioprotective effect of RES, which was manifest in lowered left ventricular peroxidation and enhanced antioxidant production, such as GSH and SOD, as well as, reduced TNF-α levels (304) (**Table 1** and **Figure 2**). Another study showed RES inhibition of TLR4/NF-κB signaling in an ischemic injured rat heart model, which is confirmed by TLR4 and NF-κB downregulation, and reduced myocardial TNF-α production (303) (**Table 1** and **Figures 1** and **2**).

**Table 1:** The anti-inflammatory mechanism(s) of phytochemicals and their associated epigenetic modifications/effect.

	Origin	Anti-inflammatory mechanism	Epigenetic modifications	Epigenetic modification effect
**RES**	phenolic stilbene derivative obtained from grape skins, berries and peanuts (124, 132).	It inhibits TLR4 and MyD88 expression in activated RAW 264.7 macrophages (142, 143) It inhibits NF-κB, MAPK, IRF-3 and AP-1 transcription factors, as well as, iNOS, COX-2, and 5-LOX enzymes (124, 143–147). It reduces NF-κB induced proinflammatory cytokines, including TNF-α, IL-6, and IL-1β, and the free radicals, NO and ROS, and LTs and PGs levels (124, 132, 140, 142, 147–148). It inhibits TLR4/NF-κB signaling in ischemic injured rat heart model, and reduces myocardial TNF-α production (148).	It downregulates miR-155 in RAW264.7 (149). RES downregulated miR-21 in human glioblastoma and in different *in vitro* models (150, 151). RES suppresses miR-146a in RAW 264.7 macrophages (151). RES increased DNMT 3a and 3b expression in the retinal epithelial (ARPE-19) cell line (152). RES deacetylates the promoter region of MMP9 endoproteinase. RES targets HDAC complexes (104–106)	Upregulation of SOCS1 expression, and inhibits the inflammatory factors, TNF-α, IL-6, MAPKs (149). It modulates AP-1 activity in THP-1 human monocytes (150) Reduction in IkB phosphorylation and NF-κB activity (150). Modulation of Nfr2 in LPS-stimulated macrophages (151). RES-mediated DNA hyper-methylation reversed oxidative stress and inflammation-dependent changes (152). Downregulation of MMP9 expression, and suppress inflammation-induced tissue remodeling (153, 154). Regulation of JNK and NF-κB activity.
**CUR**	A polyphenolic-yellow pigment that is obtained from turmeric *(Curcuma longa)* (155, 156).	It inhibits lipid peroxide formation and lysosomal enzymes (157, 158). It attenuates oxidative stress during inflammation by activating the Nrf2-Keap1 pathway and increasing the activity of antioxidant enzymes (159). It increases the activity of serum antioxidants (e.g. SOD and GSH), and it scavenges ROS and RNS (156, 160–163). It inhibits iNOS, 5-LOX, COX-1 and COX-2 (132). It modulates TLR4 and MyD88 pathways in macrophages *via* blocking NF-κB activation (164, 165). It inhibits MAPK and AP-1 activation and IκB-α phosphorylation (166, 167). It binds non-covalently to MD-2 and inhibits both MyD88-dependent and TRIF-dependent pathways (168–170). It inhibits M1 macrophage polarization by TLR4 downregulation (171). It inhibits TNFα, IL-1β, and IL-6 proinflammatory cytokines, as well as, ICAM-1 cell adhesion molecule (124, 147, 171, 172).	It downregulates miR-155 in LPS-induced RAW 264.7 macrophages (173, 174). It reduced miR-21 and miR-155 in clinical studies. CUR inhibits p300 HAT in CVD experimental models (175–177). It inhibits HDAC I activity in cardiac cells (178). It inhibits HDAC I and HDAC III (179). It inhibits DNMTs in non-alcoholic fatty liver disease (180, 181).	Degradation of PI3K/AKT pathway (174) Suppression of AKT and JNK proliferation kinases, AP-1 transcription factor, and decreased NF-κB activation, TNF-α and IL-6 synthesis (182, 183). Reduction of histone acetylation on the promoter regions of GATA4, and suppression NF-κB-dependent inflammation. Increasing TIMP1 gene expression, downregulating MMP2, and attenuating cardiac fibrosis and inflammation (178). It suppresses NF-κB activity in human hematopoietic Raji cells (179). It represses DNA hypermethylation at PPARα promoter and thus upregulates PPARα expression and reduced liver cell death (180, 181).
**Quercetin**	A plant flavonoid, quercetin found in citrus fruits, apples, onions, red grapes and tea (184, 185).	It negatively regulates LPS-induced TLR4 expression and signaling, prevents NF-κB translocation, and inhibits COX-2 and iNOS expression in macrophages and human PBMCs (166, 186, 187). It significantly reduces proinflammatory cytokines production by suppressing the activation of ERK and p38 MAP kinase, and NF-κB/IκB signaling pathways in LPS-activated macrophage (188). It inhibits NF-κB pathway *via* activating Nrf2 signal transduction cascade (120).	It decreases miR-155 expression (120, 189). It increases miR-146a expression. It attenuates p300/HAT-mediated signaling in breast cancer cells attenuating (190).	Inhibition of NF-κB activation, and downregulation of TNF-α, IL-6, and IL-1β proinflammatory cytokines (120, 189). Reduction of NF- κB, and downregulation of TNF-α, IL-6 and IL-17 (151, 191, 192). Suppressing COX-2 expression, and showing a protective effect against inflammation-dependent cancer (190).
**API**	A plant-derived flavonoid abundant in many fruits and vegetables, including parsley, celery, and chamomile tea (193, 194).	It reduces the levels of NO, TNF-α, IL-6, IL-1β and PGs *via* inhibiting iNOS, NF-κB and COX-2 activity in several *in vitro* and *in vivo* LPS-induced inflammation models (148). It reduces oxidative stress, downregulates the TLR4/NF-κB signaling pathway, decreases IL-6 and TNF-α levels, and inhibits mitochondria-mediated neuron apoptosis (195). It acts as potent M1–M2 modulator in adipose tissue macrophages by blocking the inflammatory processes *via* PPARγ, and it suppresses obesity-related inflammation (196, 197). It inhibits COX-2 and NF-κB gene expression in LPS-mediated acute lung injury (198).	It downregulates miR-155 by inhibiting NF-κB In LPS-induced macrophages (199). It decreases the expression of DNMT1, DNMT3a, DNMT3b, as well as, some HDACs (200).	Upregulation of an NF-κB inhibitor, FOXO3a and TNF-α suppressor, SMAD2 (201, 202). It increased Nrf2 mRNA and protein expression in JB6 P+ skin epidermal cells (200).
**Genistein**	An isoflavonoid obtained from soy-based foods, red clover and legume (203).	It prevents endothelial inflammatory damage by blocking NF-κB and downregulating IL-6, and ICAM-1 (204). It reverses angiotensin II-induced atherosclerotic inflammation through suppressing the expression of NF-κB, and the phosphorylation of ERK1/2 and p-38 (205). It hinders TLR4 dimerization, abolishing MyD88 or TRIF dependent pathways and inactivating NF-κB, which downregulates proinflammatory cytokines (206). It activates Nrf2 pathway, accounting for its antioxidant activity (206). It suppresses LPS-induced NF-κB activation by targeting AMPK in macrophages (207).	It suppresses miR-155/SOCS1 (208).	Inhibition of NF-κB signaling and Reversal of ox-LDL-induced inflammation in HUVECs cells (208).
**EGCG**	A catechin- polyphenol, mainly found in green tea, onions, apple skins, and plums (209).	It suppresses LPS-induced TLR4 signaling, and reduces the receptor expression (210). It activates NRF2, and protect cells from inflammation-induced oxidative stress (211). It attenuates airway inflammation by reducing immune cells infiltration and induced levels of TNF-α, IL-2, and IL-6 in asthmatic mice (212). It protects neuronal cells from microglia-induced cytotoxicity by suppressing amyloid β-induced TNFα release (213).	It regulates p300 HAT and HDACs I and II differential binding at promoter regions of NF-κB subunit p65 gene (214).	Decrease in proinflammatory genes expression in stress-induced endothelial cells, and reduces atherosclerosis and fibrogenesis (214–216).
**Emodin**	An anthraquinone compound that is abundant in buckthorn, knotweed and rhubarb (217).	It inhibits induced TLR4, MyD88 and TRAF6 expressions in inflammatory pneumonia model, and decreases p38/JNK MAPK phosphorylation and NF-κB p65 nuclear translocation, yet activates Nrf2 pathway, thereby suppressing inflammation (218). It blocks the nuclear translocation of STAT1, IRF5, and NFκB-p65 in M1 macrophages, while inhibits STAT6 and IRF4 in M2 macrophages (219). It restores the balance between M1 and M2 hyperpolarization in macrophages (219).	It increases H3K27 trimethylation at the promoter regions of iNOS, TNF-α, IL6 and IRF4 in macrophages (219). It decreases HDAC I and II activity and increases histone acetylation (220).	Downregulation of iNOS, TNF-α, IL6 and IRF4 in activated macrophages (219). It blocks NF-κB in cardiac myocytes through HDAC inhibition and increasing histone acetylation (220, 221), It blocks pyroptosis by attenuating NOD-, LRR- and NLRP3 inflammasome pathway in hypoxic-induced heart cells (221). It prevents cardiac dysfunction in pre-clinical animal models of heart failure (222–225).
**ACNs**	A flavonoid found in berries, grapes, and potatoes (27).	ACNs ameliorate neuroinflammation by decreasing TLR4 expression and inactivating NF-κB, reducing proinflammatory mediators, such as iNOS and TNF-α (226). They inhibit oxidative stress by activating the Nrf2/HO-1 signaling pathway (227). They attenuate fatty liver and inflammation (228, 229). They enhance metabolic activity (230–232).	They induce histone H3 acetylation at lysine residues K9, K14 and K18 in fibrosis-related genes in liver. They modulate HDAC and HAT activity (233–235)	Decrease in liver fibrosis in rats exposed to carbon tetrachloride (236, 237). They attenuate proinflammatory TNF-α signaling and gene expression in mice liver (238).
**EA**	A polyphenolic compound widely spread in fruits, including raspberries and strawberries, mushrooms, and nuts (239).	EA reduces inflammatory response and oxidative stress by inhibiting TLR4 and activating Nrf2 (240). It reverses inflammation and adiposity (241–244) by mitigating the activity of NF-κB (243). It attenuates adipogenesis and adipocyte function by suppressing PPAR- γ (245–248).	It inhibits the activity of CARM1 methyltransferase enzyme (249–251). It attenuates differentiation-induced hyper-demethylation of histone 3 arginine 17 in human adipose-derived stem cells (245).	It reduces inflammation processes mediated by either NF-κB or metabolic dysfunction (249–251). It attenuates excess adipose tissue accumulation and downstream inflammation and metabolic impairment (252).
**Tanshinone IIA**	A diterpenoid, extracted from the root of *Salvia miltiorrhiza* Bunge (Danshen) (253).	It suppresses p38 MAPK signaling pathway, and reduces arrhythmogenesis following myocardial infarction, and enhances cardiac function (254, 255). It inhibits the expression of TLR4, MyD88, GM-CSF, IL-1β, TNF-α, and COX-2, and attenuates LPS-mediated TLR4-NF-κB pathway activation (256, 257).	It reduces miR-155 expression (256–258). It inhibits over-expressed miR-146 and miR-155 (259). It targets miR-155 induced levels in LPS-induced macrophages (260). It inhibits the protein levels of DNMT1, DNMT3a, DNMT3b, as well as, HDAC3 and HDAC activity, at Nrf2 promoter and reduces its methylation (261).	Reduction in LPS-induced inflammation process. Relative inhibition of the levels of inflammatory markers, namely CRP, ox-LDL, CCL-2, CD40, and MMP-2, as well as the proinflammatory cytokines, IL-1β, IL-6, IL-12, and TNF-α in *gingivalis-*induced atherosclerosis (259). It suppresses the proliferation of inflammation-induced colon cancer cells (260). It increases Nrf2 expression and its downstream targets, and inhibits TPA-induced JB6 cells transformation (261).
**Carvacrol Thymol**	Monoterepnoids derived from the essential oil of *Origanum vulgare* L. or wild bergamot (253).	Car/Thy reduce the activation of TLR4/NF-κB signaling pathway, whereas increase SOD1 and GSH antioxidants through Nrf2 Activation, attenuating oxidative damage (262, 263). They suppress allergic inflammation associated with asthma	Car/Thy downregulate miR-155, miR-146a, and miR-21 (264),.	They reduce TLR4 induced expression and reverse miRNA-mediated suppression of SOCS1 and SHIP1 negative regulators (264).
**BAs**	Active ingredient derived from boswellic acids that are extracted from oleo-gum-resin of *Boswellia serrata* (265, 266).	It attenuates LPS-induced neuroinflammation (265). They downregulate TLR4 receptor and MyD88 expression, and suppress NF-κB p65 and p-JNK in hepatotoxicity (267). BAs upregulate Nrf2 and HO-1 expression, thereby protect liver from induced oxidative injury (268). BAs exhibit neuroprotective effect by modulating Nrf2/HO-1 pathway (269).	They reduce miR-155 expression levels in chronic inflammatory disorders (265).	They suppress IκB-α expression levels, whereas increases SOCS-1, resulting in decreased apoptotic activity and amyloid protein genesis, and eventually attenuate chronic inflammation (265).
**SFN**	An isothiocyanate compound hydrolyzed from its precursor, glucoraphanin, and found in cruciferous vegetables from the Brassicaceae family, including broccoli, cabbage, cauliflower, and kale (270, 271)	It targets monocytes/macrophages lineage and stimulates Nrf2 pathway in chronic inflammatory diseases (272, 273). It activates Nrf2 pathway, and reduces NF-κBNF-κB expression and AP-1, thus restoring endogenous antioxidant levels and reducing inflammatory damage in autoimmune encephalomyelitis mice model (274–279). It acts as an indirect antioxidant, and upregulates some phase II enzymes by enhancing Nrf2 activity (271, 280–283). It suppresses the direct binding between NF-κB and its DNA consensus sequence, and thus suppresses LPS-induced levels of TNF-α, iNOS, and COX-2 in macrophages (168, 271, 284–286). It suppresses both ligand-induced and ligand-independent oligomerization of TLR4 in macrophages (287). It antagonizes LPS binding to TLR4/MD-2 complex by selectively competing on MD-2 (168, 284, 285, 287, 288). It prevents inflammation-related carcinogenesis (274).	It downregulates induced miRNA-155 and 146a levels in LPS-stimulated RAW264.7 macrophages (289, 290). It targets DNA methylation (291–295). It targets DNMT, and suppresses mediated-DNA hypermethylation at Nrf2 promoter region (296).	Suppression of LPS-induced inflammation in macrophages and NF-κBNF-κB signaling attenuation (289, 290). Inhibition of Inflammation, and dependent chemopreventive effects (291–295). Increased Nrf2 expression and subsequently, decreased neurological inflammation, as well as inflammatory-associated cytokines, IL-6 and IL-1β (296).
**AITC**	An isothiocyanate derived from its precursor sinigrin, and is abundant in different brassica species such as mustard, wasabi, and horseradish (120).	It enhances the nuclear translocation of Nrf2, and represses the expression of NF-κB; subsequently, it upregulates HO1 levels, and suppresses inflammation (120).	It represses miR-155 levels in murine RAW 264.7 macrophages (120).	Modulation of NF-κB and Nrf2 signaling pathways, and lowering of induced levels of iNOS, TNF-α, and IL-1β, thus attenuating inflammation (120, 289)
**CA**	A conjugated aromatic aldehyde isolated from *Cinnamomum cassia* Presl bark (253).	It decreases NF-κB activity, and downregulates the levels of COX-2 and iNOS, and the proinflammatory cytokines, TNF-α, IL-1β, and IL-6, and other factors, such as ROS, NO, and PCs; in addition to NLRP3 inflammasome, and thus mitigates inflammation symptoms in macrophages and different *in vitro* and *in vivo* LPS-induced inflammation models (147, 148, 253). It disrupts TLR4/MD-2 heterodimer *via* covalent adducts formation (169, 297). It inhibits LPS-induced oligomerization of TLR4 receptor (298). It activates Nrf2 pathway (299).	It decreases the expression of miR-155 and miR-21 in macrophages (253).	Suppression of IL-1β and IL-6 inflammatory markers in macrophages, and dependent-protection against ulcerative colitis (300).

Through epigenetic mechanisms (**Table 1** and **Figure 3**), RES attenuated LPS-mediated inflammation in RAW264.7 macrophages through downregulating miR-155 and concurrently boosting, SOCS1 expression, leading to the inhibition of the inflammatory factors, TNF-α, IL-6, MAPKs (149). Additionally, Tili et al. showed another mechanism by which RES modulates AP-1 activity in THP-1 human monocytes through downregulating miR-155 (150). RES also downregulated miR-21 in different *in vitro* models (151). Li et al. introduced the inhibitory effect of RES on miR-21 expression in human glioblastoma (U251) cells, leading to a reduction in IκB phosphorylation and NF-κB activity (305). In RAW 264.7 murine macrophages, Bigagli et al. depicted the suppressive effect of RES on miR-146a, which targets the transcription factor nuclear factor (erythroid-derived 2)-like 2 (Nrf2) responsible for inhibiting proinflammatory mediators. Nfr2 was also positively modulated by the RES in LPS-stimulated macrophages (306). These findings indicate that RES modulatory effect on these miRNAs can be regarded as a buffering effect against physiological imbalance. Besides miRNAs, RES caused an increased DNMT activity, especially in DNMT3a and DNMT3b expression in the retinal epithelial cell line, ARPE-19 (152). This RES-mediated DNA hypermethylation results in a reversal of oxidative stress and inflammation-dependent changes (152). In addition to regulation of NF-κB and MAPKs signaling cascades, RES could inhibit inflammation through regulating histone deacetylation-dependent gene expression (153, 307). RES deacetylates the promoter region of matrix metalloproteinase 9 (MMP9), which in turn downregulates the expression of MMP9, an endoproteinase that is involved in inflammation-induced tissue remodeling and is activated by MAPK, c-Jun N-terminal kinases (JNK), and NF-κB binding (153, 154). Furthermore, independent of its histone deacetylase activity, RES could attenuate MMP9 activity, because this stilbene is known to inhibit JNK and NF-κB (308, 309). Moreover, JNK and NF-κB have been shown to be regulated by HDAC complexes, which suggests that RES can also regulate the activity of these molecules by acting on HDACs (104–106). Although RES showed positive outcomes in animal studies time and again, these results have not been well translated in clinical trials (27).

#### Curcumin

Curcumin (CUR) is another naturally polyphenol. It is found as a yellow pigment that is obtained from turmeric *(Curcuma longa)*, a flowering plant of the ginger family (155, 156). Traditionally, CUR is widely used in food as a curry coloring or flavoring spice (132). The phenolic compound exerts diverse bioactive effects; it has anti-bacterial, anti-inflammatory, antioxidant and anticancer functions (124, 310–313). Although CUR has a great many therapeutic benefits. Its main advantage lies in its antioxidant and anti-inflammatory effects (314, 315). CUR supplements are usually taken three times per day, with a dosage of 400–600 mg, and even with no side effects on kidney or liver when taken up to 12 g/day (316, 317). Only at very high doses, it may cause stomach upset with extended use, and gastric ulcers (132). CUR is usually combined with enhancing agents, such as piperine, to overcome its poor absorption and low bioavailability (318–320). Over 100 clinical trials on the anti-inflammatory activity of CUR have been published in PubMed, showing the significance of CUR in multiple inflammatory disorders, such as rheumatoid arthritis, inflammatory bowel diseases, nephropathies, and some cancers (124, 314).

##### CUR Mechanisms of Action as an Anti-Inflammatory Agent

CUR has a pleiotropic mechanism of action against inflammation (157) (**Table 1**, **Figures 1** and **2**). Firstly, it has powerful antioxidant activity that inhibits lipid peroxide formation and lysosomal enzymes, such as acid phosphatase and cathepsin D (157, 158). CUR attenuates oxidative stress during inflammation by activating the Nrf2-Keap1 pathway and increasing the activity of antioxidant enzymes (159) (T1 & **Figures 1** and **2**). By increasing the activity of serum antioxidants such as superoxide dismutase (SOD) and glutathione peroxidase (GSH), CUR scavenges different free radicals, including reactive oxygen and nitrogen species (ROS and RNS), and peroxyl radicals (156, 160–163). CUR can also inhibit different ROS-generating enzymes, such as iNOS, COX system (COX-1 and COX-2), and 5-LOX, and suppresses the activity of several PGs (132) (**Table 1** and **Figure 2**). Therefore, CUR can be considered a natural alternative to NSAIDs for inflammation (132, 140, 156). Secondly, CUR has a potent anti-inflammatory effect that derives from its modulation of TLR4 and MyD88 pathways in macrophages and evidenced by blocking NF-κB activation (164, 165) (**Table 1** and **Figures 1** and **2**). CUR inhibits the activation of MAPK and AP-1 transcription factors, and also IκB-α phosphorylation and degradation (166, 167) (**Table 1** and **Figures 1** and **2**). On top of that, CUR has been suggested to non-covalently bind MD-2 (a lymphocyte antigen responsible for LPS binding to TLR-4), which results in a competition with LPS for the TLR4/MD-2 complex that leads to the inhibition of both MyD88-dependent and TRIF-dependent pathways (168–170) (**Table 1** and **Figures 1** and **2**). Interestingly, CUR inhibited M1 macrophage polarization in a dose-dependent manner through downregulating the expression of TLR4 (171) (**Table 1** and **Figure 1**). These antagonistic effects to TLR4 signaling pathways and its downstream mediators are followed by an inhibition of proinflammatory cytokines, including TNFα, IL-1β, and IL-6 (124, 147, 171, 172) (**Table 1** and **Figure 2**). Research studies suggest that CUR pretreatment protects against T cell-mediated hepatitis in mice and that the significant effect of CUR may be partly through inhibiting the expression levels of TLR2, TLR4 and TLR9 in the liver (321). Furthermore, CUR blocks the expression of cell adhesion molecules, such as ICAM-1, which are involved in the interaction between leukocytes and endothelial cells (124, 322) (**Table 1**).

Post-transcriptionally (**Table 1** and **Figure 3**), on the miRNA level, several studies on RAW 264.7 macrophages revealed the significant inhibitory effect of CUR on miR-155, which is a key transcriptional regulator of TLR4-mediated inflammatory reactions (173, 174). *In vivo* and *in vitro* reports confirmed the downregulation of miR-155 expression by CUR through degrading phosphoinositide 3-kinase PI3K/AKT pathway after LPS stimulation (174). In clinical studies, daily intake of CUR decreased miR-21 and miR-155 expression. This decrease was followed by a suppression of AKT and JNK proliferation kinases, and the transcription factor AP-1, which attenuated inflammation *via* decreased NF-κB activation, TNF-α and IL-6 synthesis (182, 183).

At the epigenetic level, CUR exhibits pleiotropic mechanisms to regulate multiple molecular targets (27) (**Table 1** and **Figure 3**). Knowing that NF-κB activity is regulated, in part, through p300 HAT-dependent actions (104). One target involves the regulation of histone acetylation by CUR-mediated p300 inhibition in CVD experimental models (175–177). This inhibition reduces histone acetylation on the promoter regions of the cardio pro-hypertrophic gene, GATA binding protein (GATA4), a protein which mediates inflammation through NF-κB -dependent mechanisms (177, 323, 324). Besides its HAT inhibitory actions, CUR has also been involved in the inhibition of inflammation-induced cardiac remodeling through inhibiting HDACs (178). Through inhibiting HDAC1 activity and thus, increasing histone acetylation at the promoter region of tissue inhibitor of metalloproteinase 1 (TIMP1), CUR increased TIMP1 gene expression and, therefore, attenuated cardiac fibrosis and inflammation (178). By increasing the expression of TIMP1, CUR reduced the expression of the TIMP1 inhibitory target, called metalloproteinase 2 (MMP2), which contributes to inflammatory signaling (178, 325). Another study by Marquardt and colleagues revealed that CUR inhibits the activity of NF-κB in human hematopoietic Raji cells *via* inhibition of histone deacetylase, HDAC1 and HDAC3 (179). In addition to histone acetylation, CUR was shown to repress DNA hypermethylation at CpG sites within the promoter region of peroxisome proliferator-activated receptor-alpha (PPARα) by inhibiting DNMTs in a non-alcoholic fatty liver disease, which results in upregulated PPARα expression and reduced liver cell death (180, 181)., PPARα, a transcription factor that predominates in the liver, regulates the expression of genes involved in inflammation and several metabolic processes (326–330).

#### Quercetin

A plant flavonoid, quercetin (3, 3′, 4′, 5, 7-pentahydroxyflvanone) is found in citrus fruits, apples, onions, red grapes and tea (184, 185). This phenolic compound exhibits an anti-inflammatory, antioxidant, chemopreventive and neuroprotective properties (331). The estimated dosage ranges from 50 to 800 mg/day, which is mainly dependent on dietary habits (332). The anti-inflammatory potential of quercetin can be discerned in different cell types of both animal and human models (333). Quercetin plays a modulatory, biphasic and regulatory action in inflammation and immunity, and possesses an immunosuppressive effect on dendritic cell function (334, 335).

##### Quercetin Mechanism of Action as an Anti-Inflammatory Agent

Quercetin negatively regulates LPS-induced TLR4 signaling (**Table 1**, **Figures 1** and **2**). It reduces TLR4 expression and prevents NF-κB translocation to the nucleus in macrophages and human PBMCs, thereby ameliorating the inflammatory response (166, 186, 187, 336). Also, it inhibits COX-2 and iNOS gene expression *in vitro*, and significantly reduces the production of proinflammatory cytokines *via* MAP kinases and NF-κB pathway in LPS-activated macrophages (188). In this highlight, Cho et al. show that this flavonoid suppressed the activation of phosphorylated ERK kinase and p38 MAP kinase (not JNK MAP kinase), and inhibited NF-κB activation by stabilizing the NF-κB/IκB complex in LPS-treated RAW 264.7 macrophages (188).

Few reports have addressed the modulation of inflammatory-related miRNAs by quercetin (184) (**Table 1** and **Figure 3**). The effect of quercetin and its main metabolites on miR-155 have been evaluated in LPS-stimulated macrophages. It has been reported that quercetin and its metabolite, known as isorhamnetin, downregulate the mRNA and protein levels of the proinflammatory mediators, including TNF-α, IL-6, and IL-1β by decreasing the expression of miR-155, which represents a mechanism by which this polyphenol may inhibit the activation of NF-κB, contributing to the containment of the inflammatory process (120, 189). These two compounds can also indirectly inhibit the NF-κB pathway through activating the Nrf2 signal transduction cascade (120) (**Table 1** and **Figure 2**). Nonetheless, the direct activity of miR-155 on Nrf2 signaling remains a promising area for further investigations in order to determine whether Nrf2 signaling might be directly affected by miR-155. In this context, Saadatmandi et al. revealed that miR-155 targets Bach1, a Nrf2 signaling repressor (189). Furthermore, quercetin has been shown to upregulate miR-146a, which is followed by a reduction in the levels of NF-κB and the downregulation of TNF-α, IL-6 and IL-17 (151, 191, 192). Additionally, quercetin’s protective effect on inflammation-dependent cancer was evident in its suppression of COX-2 in breast cancer cells *via* its attenuation of p300/HAT-mediated signaling (190) (**Table 1** and **Figure 3**).

#### Apigenin

Apigenin (API, 4’,5,7-dihydroxyflavone) is a plant-derived flavonoid abundant in many fruits and vegetables, including parsley, celery, and chamomile tea (193, 194). This compound is widely used as an anti-inflammatory agent, and it also has anticancer and cardioprotective properties (337, 338).

##### Apigenin Mechanism of Action as an Anti-Inflammatory Agent

In different *in vitro* and *in vivo* inflammation models induced by LPS, API causes a reduction in the levels of NO, TNF-α, IL-6, IL-1β and PGs through the inhibition of iNOS, NF-κB and COX-2 activities (148) (**Table 1** and **Figures 1** and **2**). A number of recent studies have highlighted the importance of API as a potent M1/M2 modulator, downregulating NO production and proinflammatory cytokines (197, 339). Favoring M2 polarization, API can also block the inflammatory processes in adipose tissue macrophages through PPARγ (196) (**Table 1**). In obese animal models, it plays a role in suppressing obesity-related inflammation (197). Balex et al. showed that API exerts its anti-inflammatory activity in LPS-mediated acute lung injury through inhibiting the gene expression of COX-2 and NF-κB (198) (**Table 1** and **Figures 1** and **2**). Whereas, Zhao et al. revealed API effect in neuro-inflammation; it reduces oxidative stress, downregulates the TLR4/NF-κB signaling pathway, decreases the levels of IL-6 and TNF-α, and inhibits mitochondria-mediated neuron apoptosis (195) (**Table 1** and **Figures 1** and **2**). In LPS-induced macrophages, miR-155 is downregulated by API treatment through inhibiting NF-κB (199) (**Table 1** and **Figure 3**). Another study showed that succeeding miR-155 suppression by API, an observed upregulation of two miR-155 targets, namely FOXO3a (Forkhead Box O3a), an inhibitor of NF-κB, and SMAD2 (smooth-muscle-actin and MAD-related proteins 2), a suppressor of TNF-α and iNOS inflammatory molecules (199, 201, 202). In addition to miRNA-related epigenetic modifications, API successfully demethylated the promotor region of Nrf2, resulting in an increased Nrf2 mRNA and protein expression in JB6 P+ skin epidermal cells. This effect was mediated through a reduction in the expression of the epigenetic proteins, DNMT1, DNMT3a, DNMT3b, as well as some HDACs (200) (**Table 1** and **Figure 3**).

#### Genistein

An isoflavonoid, Genistein is primarily obtained from soy-based foods, red clover and legumes (203). Amongst many isoflavones, genistein is widely recognized for its antioxidant and anti-inflammatory functions, as well as, its anticancer and anti-proliferative activity (340). This compound has been successfully utilized as an immunosuppressive agent *in vitro* and *in vivo* (341). Unfortunately, the oral bioavailability of genistein and its plasma concentrations were very low *in vivo*, which might affect its efficacy, and interfere with the consistency of its pharmacological results in clinical trials (342–345).

##### Genistein Mechanism of Action as an Anti-Inflammatory Agent

Genistein prevented endothelial inflammatory damage by blocking NF-κB and the expression of the proinflammatory cytokine, IL-6, and adhesion molecule, ICAM-1 (204) (**Table 1** and **Figures 1** and **2**). A recent study by Xu et al. highlighted the inhibitory effect of genistein on angiotensin II-induced vascular smooth muscle cell inflammation, in which angiotensin II induced the expression of NF-κB, C-reactive protein (CRP), and the phosphorylation of ERK1/2 and p-38, leading to atherosclerotic inflammation, which is reversed after genistein treatment (205) (**Table 1** and **Figures 1** and **2**). Moreover, this isoflavonoid compound enhanced PPAR-γ expression, displaying a cardiovascular protective property *via* the regulatory crosstalk between p38/ERK1/2-PPARγ-NFκB signaling pathways (205). Another study linked the inhibitory effect of Genistein on LPS-mediated NF-κB activation in macrophages to the activation of adenosine monophosphate kinase (AMPK), which lead to repression of inflammation (207) (**Table 1**). Besides, Genistein is involved in hindering TLR4 dimerization, and thus abolishes MyD88 or TRIF dependent pathways, inactivating NF-κB and inhibiting its translocation into the nucleus, which, in turn, prevents proinflammatory cytokines transcription (206) (**Table 1** and **Figures 1** and **2**). In line with its anti-inflammatory properties, Genistein possesses notable anti-oxidant activities *via* activating Nrf2/NQO1 pathway (206) (**Table 1** and **Figure 2**).

Similar to the previously mentioned polyphenols, genistein plays a role in modulating TLR4-responsive miRNAs. Through miR-155/SOCS1-mediated suppression of the NF-κB signaling, genistein reversed ox-LDL-induced inflammation in human umbilical vein endothelial cells (HUVECs) (208) (**Table 1** and **Figure 3**).

#### Epigallocatechin-3-Gallate (EGCG)

One of the most popular polyphenolic catechinsis EGCG, which is mainly found in green tea, onions, apple skin, and plums (209). There is an emerging group of evidence on its biological activity where it was found to exert remarkable anti-inflammatory, antioxidant, anticancer, and antiangiogenetic effects (346–350). A number of reports introduced the prominent anti-inflammatory effect of EGCG (351).

##### EGCG Mechanism of Action as an Anti-Inflammatory Agent

EGCG has been reported to suppress LPS-induced TLR4 signaling, and to reduce the receptor expression (210) (**Table 1** and **Figures 1** and **2**). A potent activator of Nrf2, EGCG plays a critical role in inflammation-induced oxidative stress (211) (**Table 1** and **Figure 2**). A recent study showed that EGCG attenuated airway inflammation in asthmatic mice by significantly reducing asthmatic symptoms, including inflammatory cell infiltration, and inflammatory induced levels of TNF-α, IL-2, and IL-6 (212). Another report highlighted the cytoprotective role of EGCG on neuronal cells against microglia-induced cytotoxicity and in suppressing amyloid β-induced TNF-α release  (213).

Epigenetically, EGCG was also reported to mediate inflammation by regulating histone acetylation (214) (**Table 1** and **Figure 3**). For example, a study by Liu and colleagues showed that EGCG regulated p300 and HDACs I and II differential binding at promoter regions of the NF-κB subunit p65 gene, and, consequently, decreased proinflammatory gene expression in stress-induced endothelial cells (214). It should be duly noted that endothelial cells may become impaired due to persistent inflammation, resulting in atherosclerosis and fibrogenesis (215, 216). Since NF-κB inflammatory action is dependent on p300-, HDAC1- and HDAC2-mediated actions (103, 104), EGCG, therefore, successfully prevents inflammation in endothelial cells *via* regulating histone acetylation at proinflammatory gene promoters (214). Collectively, this data outlines the anti-inflammatory activity of EGCG in regulating both inflammation homeostasis and cardiac function *via* histone acetylation-dependent mechanisms.

#### Emodin

Emodin is an anthraquinone that is abundant in plants, such as buckthorn, knotweed and rhubarb (217). As a traditional Chinese medicine, emodin has been used for viral and bacterial infections, and for kidney and gastrointestinal disorders (27).

##### Emodin Mechanism of Action as an Anti-Inflammatory Agent

Emodin significantly inhibits induced TLR4, MyD88 and TRAF6 expressions in inflammatory pneumonia model, and decreases p38/JNK MAPK phosphorylation and NF-κB p65 nuclear translocation, whereas it activates Nrf2 pathway, thereby suppressing inflammation (218) (**Table 1** and **Figure 1**). Emodin also blocks the nuclear translocation of signal transducer and activator of transcription 1 (STAT1), IRF5, and NF-κB-p65 in M1 macrophages, whereas it inhibits STAT6 and IRF4 in M2 macrophages (219). Macrophage hyperpolarization into either M1 or M2 phenotype is detrimental (352, 353). Most phytochemicals usually target M1 hyperpolarized macrophages. However, emodin is able to restore the balance between M1 and M2 hyperpolarization through epigenetic balancing of macrophage activation (219).

At the epigenetic level (**Table 1** and **Figure 3**), recent reports showed that emodin increases H3K27 trimethylation in activated macrophages (219). This was evident at the promoter regions of the inflammatory signaling genes, iNOS, TNF-α, IL-6 and IRF4 in macrophages, to the end result of a reduction in their expression (219). Pertaining to its chelating properties, emodin is considered an HDAC inhibitor that chelates zinc ions within HDAC catalytic domains, thus regulating proinflammatory signaling cascades (354). It, therefore, increases histone acetylation by decreasing HDAC I and II activities (220). For instance, emodin was recently shown to block the proinflammatory signaling molecule, NF-κB, and pyroptosis in cardiac myocytes through HDAC inhibition, since NF-κB activity is regulated by HDACs (220, 221). In this regard, HDAC inhibition has been shown to prevent/reverse cardiac dysfunction in pre-clinical animal models of heart failure (222–225). Through this mechanism, emodin successfully attenuated NOD-, LRR- and pyrin-domain containing protein 3 (NLRP3) inflammasome pathway in hypoxic-induced heart cells (221), noting that NLRP3 inflammasome is involved in the synthesis of proinflammatory byproducts, and mediating inflammation-induced cell death or pyroptosis (355).

#### Anthocyanins

Flavonoids with three phenolic rings, anthocyanins(ACNs) are involved in food pigmentation, and are found in berries, grapes, and potatoes (27). Recently, anthocyanin-rich foods have proved their metabolic efficacy in humans (230–232), and have been effectively used to alleviate fatty liver and inflammation (228, 229).

##### Anthocyanins Mechanism of Action as an Anti-Inflammatory Agent

Notably, ACNs ameliorate neuroinflammation by decreasing TLR4 expression and inactivating NF-κB, reducing proinflammatory mediators, such as iNOS and TNF-α (226). They also inhibit oxidative stress by activating the Nrf2/HO-1 signaling pathway (227) (**Table 1** and **Figure 1**).

Not only this, but also ACNs exhibit epigenetic modulation capacity (**Table 1** and **Figure 3**). Protecting the liver by mediating changes in histone acetylation, anthocyanin-rich extract induced histone H3 acetylation at lysine residues K9, K14 and K18 and decreased liver fibrosis in rats exposed to carbon tetrachloride (236, 237). Indeed, K9 and K14 acetylation is essential for proper liver function (356). Therefore, anthocyanins were able to decrease liver fibrosis by regulating gene expression through histone acetylation. Another study showed that ACNs modulate HDAC and HAT activities (233–235). However, the link between anthocyanin-mediated histone acetylation and HDAC or HAT activities remains unclear (236, 237). Notably, increased HAT activity and therefore, hyperacetylation of H3K9/14 at the promotor site of TNF-α, was shown to be associated with liver inflammation, leading to fibrosis in obesity-induced mice (357). That’s why most phytochemicals exhibiting hepatoprotective properties are dependent on HAT inhibition (358). In this context, ACNs have been reported to attenuate proinflammatory TNF-α signaling and gene expression in murine livers (238). Not only this, but also they decrease HAT activity and, hence, TNF-α signaling outside the liver (233). Nonetheless, future research should be directed towards ACNs blocking effects of inflammation-dependent liver fibrosis *via* HAT inhibition.

#### Ellagic Acid

A polyphenolic hydroxybenzoic acid derivative, ellagic acid (EA) is widely present in fruits, like raspberries and strawberries, as well as in mushrooms, and nuts (239). Indeed, EA treatment has a potent anti-inflammatory activity; it has been shown to reverse inflammation and adiposity (241–244).

##### Ellagic Acid Mechanism of Action as an Anti-Inflammatory Agent

EA is known to reduce inflammatory response and oxidative stress by inhibiting TLR4 and activating Nrf2 (240) (**Table 1** and **Figure 1**). Besides, EA has been previously reported to inhibit coactivator-associated arginine methyltransferase-1 (CARM1) activity, which is a methyltransferase enzyme involved in metabolic dysfunction and NF-κB-mediated inflammation (249–251) (**Table 1** and **Figure 3**). Aside from CARM1 expression, EA remarkably attenuates differentiation-induced hyperdimethylation of histone 3 arginine 17 in human adipose-derived stem cells (245) (**Table 1** and **Figure 3**). Obesity and excessive adipose tissue accumulation are common triggers of downstream inflammation and metabolic impairment (252). In differentiated adipocytes, Kang et al. showed that EA treatment suppressed PPAR-γ, a CARM1 target and an important regulator of adipogenesis and adipocyte function that is partially coactivated by CARM1-mediated histone methylation (245–248). Therefore, the role of EA treatment in repressing PPAR-γ may be beneficial in the long term. In this regard, some reports have shown that the anti-inflammatory activity of EA is, in part, PPAR-γ-dependent (359). From this perspective, further research is required to determine its molecular targets, and to assess the extent of EA effectiveness in chronic inflammation. It is also imperative if we are to better understand how this polyphenolic compound exerts its anti-inflammatory actions epigenetically.

#### Terpenoids

##### Tanshinone IIA

A lipophilic diterpenoid, Tanshinone IIA (Tan IIA) is extracted from the root of *Salvia miltiorrhiza* Bunge (Danshen) (253). Traditionally, this herb was used to promote blood circulation, and a study by Shang et al. illustrated its cardioprotective actions (360, 361). Injections of sodium Tan IIA sulfonate was as an adjuvant in cardiovascular diseases in China (362).

##### Tan IIA Mechanism of Action as an Anti-Inflammatory Agent

Tan IIA treatment has been indicated to suppress the p38 MAPK signaling pathway, thus reducing arrhythmogenesis and mortality incidences following myocardial infarction, and enhancing cardiac function (254, 255) (**Table 1** and **Figure 1**). Additionally, this diterpenoid significantly inhibits the expression of several inflammatory mediators, such as TLR4, MyD88, GM-CSF, and proinflammatory cytokines, including IL-1β, TNF-α, and COX-2, thereby attenuating LPS-mediated TLR4/NF-κB pathway activation (256, 257). One of the most important mechanisms for inhibiting inflammation by Tan IIA is through reducing miR-155 expression, which is an upstream regulator in the process of inflammation (256–258) (T1 & **Figure 3**). Tan IIA successfully downregulated the levels of inflammatory factors, namely, CRP, ox-LDL, C-C Motif Chemokine Ligand 2 (CCL-2), cluster of differentiation 40 (CD40), and matrix metalloproteinase-2 (MMP-2), as well as the proinflammatory cytokines, IL-1β, IL-6, IL-12, and TNF-α that were induced by *Porphyromonas gingivalis* infection (259). This inhibitory effect of Tan IIA has been associated with a relative inhibition of over-expressed miR-146 and miR-155, thereby exerting significant anti-inflammatory and antioxidant properties, and ameliorating *P*. *gingivalis-*induced atherosclerosis (259). Another study illustrated that treatment with Tan IIA suppressed the proliferation of inflammation-induced colon cancer cells through inhibiting miR-155 levels in macrophages (260). This confirms the anti-inflammatory activity of Tan IIA *via* miRNAs, especially miR-155, which is suggested by the aforementioned studies to be a target of Tan IIA in inflammation. Nonetheless, the pharmacological activity of Tan IIA is not limited to miRNAs (363). Wang and colleagues showed that Tan IIA successfully inhibits murine skin epidermal JB6 cells transformation induced by TPA (12-O-tetradecanoylphorbol-13-acetate); this inhibition has been made possible through epigenetic regulation of the Nrf2 signaling pathway (261). Tan IIA treatment decreased methylation at Nrf2 promoter by inhibiting the protein levels of DNMT1, DNMT3a, DNMT3b, as well as, HDAC3 and HDAC activities, thus increasing Nrf2 expression and its downstream targets (261) (**Table 1** and **Figure 3**).

#### Carvacrol and Thymol

Monoterepnoids, carvacrol and thymol are isomers derived from the essential oil of *Origanum vulgare* L. or wild bergamot (253). The essential oil of *Origanum vulgare* L. was used, initially, for treating cold and heatstroke, and the bergamot was used as an anesthetic and antiemetic (253). Further research studied the anti-inflammatory activity of these two bioactive ingredients, carvacol and thymol (253).

##### Car/Thy Mechanism of Action as Anti-Inflammatory Agents

Car and Thy reduce the activation of TLR4/NF-κB signaling pathway in vivo and in vitro in inflammatory reactions, whereas increase the expression of antioxidants, such as SOD1 and GSH through Nrf2 Activation and attenuates oxidative damage (262, 263) (**Table 1** and **Figure 1**).

Furthermore, by regulating miRNAs and inflammatory factors, Car and Thy showed significant suppression of the allergic inflammation associated with asthma (**Table 1** and **Figure 3**). As illustrated by Khosravi and colleagues study, the inflammation-associated miRNAs, including miR-155, miR- 146a and miR-21 were overexpressed in a chitin-induced model, whereas SOCS1 and SHIP1 (miR-155 targets and negative regulators of TLR-mediated inflammation) are inhibited by chitin (264). Car/Thy reversed the induced expression of TLR4, as well as, miR-155, miR-146a, and miR-21, and reversed their effects on the negative regulators (SOCS1 and SHIP1) (264) (**Table 1** and **Figures 1** and **3**). According to these findings, the anti-inflammatory effects of Car/Thy have been linked to targeting TLR-responsive miRNAs. However, the direct targets of Car/Thy still need further investigations to be determined, since they are not reported clearly (253).

#### Boswellic Acids

Extracted from the oleo-gum-resin of *Boswellia serrata*, boswellic acids (BAs) are traditionally known to promote blood circulation and relieve pain. Boswellic acids contain various ingredients, among which is 3-acetyl-11-keto-β-boswellic acid (AKBA), which possesses a potent anti-inflammatory activity (265, 266).

#### Boswellic Acids Mechanism of Action as an Anti-Inflammatory Agent

BAs downregulate the expression of hepatic TLR4 receptor and MyD88, and suppress that of NF-κB p65 and p-JNK in hepatotoxicity model (267) (**Table 1** and **Figure 1**). Additionally, BAs upregulate Nrf2 and HO-1 expression, thereby protect liver from DOX-induced oxidative injury (268). Likewise, BAs also exhibit neuroprotective effect by modulating Nrf2/HO-1 pathway (269) (**Table 1** and **Figure 1**). Moreover, BAs have been reported to attenuate LPS-induced neuroinflammation, with the same effect as that of dexamethasone (265). They reduce miR-155 and IκB-α expression levels, while increasing SOCS-1, resulting in decreased apoptotic activity and amyloid protein genesis, which is, when accumulated, responsible for chronic inflammation (265) (**Table 1** and **Figure 3**). It’s worth mentioning that miR-155 regulation by BAs has been suggested to be a possible mechanism underlying the effective role of BAs in neuroinflammatory disorders. But, the exact targets of BAs remain an open question that requires further verification (253).

### Isothiocyanates

#### Sulforaphane

SFN is one of the highly studied plant-derived isothiocyanate organosulfur compounds (270). Characterized by the presence of a sulfocyanate group (N=C=S), SFN is found in cruciferous vegetables from the Brassicaceae family, including broccoli, cabbage, cauliflower, and kale (270, 271). SFN precursor, glucoraphanin is hydrolyzed to isothiocyanate by myrosinases enzymes (270, 364). Usually, SFN is used as a synthetic racemic mixture of D, L-SFN in research studies (271, 365, 366). In contrast to polyphenols, this isothiocyanate compound was reported to have relatively high bioavailability (around 80%), with oral dosage ranging from 20 to 40 mg in clinical trials (123, 274, 342, 367). Several reports have revealed its potential anti-inflammatory and antioxidant properties (274). In chronic inflammatory diseases, SFN exerts its potent immunomodulatory effect through targeting monocytes/macrophages and stimulating the nuclear factor erythroid-derived 2-like 2 (Nrf2) antioxidant defense pathway (272, 273). Clinically, over 1900 trials on SFN are published in PubMed (123).

##### SFN Mechanism of Action as an Anti-Inflammatory Agent

SFN has a dual action in modulating the redox system and immune imbalance, through interacting with Nrf2 and NF-κB signaling pathways (123) (**Table 1** and **Figures 1** and **2**). These two key transcription factors (Nrf2 and NF-κB) act both independently and dependently *via* their “cross talk”, which is not yet fully understood (123, 368). Several studies have pinpointed the crosstalk between Nrf2 and NF-κB pathways (271). For example, Li et al. showed that SFN activated the Nrf2 pathway through inhibiting Nrf2 ubiquitination, and concomitantly reduced NF-κB and AP-1 expression, thus restoring endogenous antioxidant levels and reducing inflammatory damage in an experimental autoimmune encephalomyelitis mice model (274–279). SFN is considered an indirect antioxidant, because it is not involved in quenching free radicals and ROS but, it upregulates some phase II enzymes by enhancing Nrf2 activity (271, 280–283). Moreover, the anti-inflammatory effects of SFN have been demonstrated in the form of reduced levels of LPS-induced proinflammatory mediators, such as TNF-α, iNOS, and COX-2 (168, 271, 284, 285). A recent study demonstrated, for the first time, the ability of SFN to suppress the direct binding between NF-κB and its consensus sequence in DNA *via* its thiol groups, therefore suppressing LPS-induced proinflammatory mediators in macrophages (286). Nonetheless, further mechanistic studies are recommended to investigate this cross-talk machinery in more details in different inflammatory models. In addition, one of the novel underlying anti-inflammatory mechanisms of SFN is its ability to suppress TLR4 oligomerization in a thiol-dependent manner in macrophages, where SFN formed adducts with cysteine residues in the extracellular domain of TLR4 (123, 168, 284, 285, 287). SFN suppressed both ligand-induced and ligand-independent oligomerization of TLR4 (287). Oligomerization is an important step for TLR4 activation and recruitment of adaptor molecules; therefore, the reactivity of SFN to the sulfhydryl moiety contributes to its inhibitory activities and subsequent downregulation of NF-κB activation (287). Similar to CUR, SFN antagonizes LPS binding to the TLR4/MD-2 complex by selectively competing on MD-2, a large hydrophobic pocket where LPS binds and mediates TLR4 dimerization (168, 284, 285, 287, 288). Furthermore, previous studies have shown that SFN successfully prevented carcinogenesis, which is partly attributed to its potent anti-inflammatory properties (274).

Importantly, studies on the miRNA level indicated that SFN significantly attenuated miRNA-155 and miRNA-146a levels in LPS-stimulated RAW264.7 macrophages in a dose-dependent manner (289, 290) (**Table 1** and **Figure 3**). Additionally, SFN exhibited chemo preventive effects that have been regulated, in part, through inhibiting inflammation *via* changes in DNA methylation (291–295) (**Table 1** and **Figure 3**). SFN attenuated DNMT-mediated DNA hypermethylation at the promoter region of Nrf2, thus increasing Nrf2 expression and subsequently, decreasing neurological inflammation, and inflammatory-associated cytokines, IL-6 and IL-1β (296) (**Table 1** and **Figure 3**). Hence, SFN regulates Nrf2 activity through DNA hypomethylation, resulting in blocking proinflammatory signaling.

#### Allyl-Isothiocyanate

Another aliphatic isothiocyanate, is allyl-isothiocyanate (AITC), which is obtained from its precursor sinigrin, and is abundant in different brassica species such as mustard, wasabi, and horseradish (120).

##### AITC Mechanism of Action as an Anti-Inflammatory Agent

Recent reports show that AITC enhances the nuclear translocation of Nrf2, which is known to repress the expression of NF-κB; subsequently, these actions upregulate HO1 (Nrf2 target gene) that further suppresses inflammation (120) (**Table 1** and **Figures 1** and **2**).

Post-transcriptionally, AITC was reported to attenuate inflammation in murine RAW 264.7 macrophages by repressing miR-155 levels, and thus lowering target proinflammatory mediators, such as iNOS, TNF-α, and IL-1β (120) (**Table 1** and **Figure 3**). Another study by Wagner et al. suggested that AITC exerted its anti-inflammatory actions *via* targeting miR-155, which acts on NF-κB and Nrf2 signaling pathways (289) (**Table 1** and **Figure 3**). In spite of this, the link between the miR-155/NF-κB/Nrf2 signaling pathway and AITC treatment remains questionable, therefore requiring future verification (289). Although Nrf2 can also be regulated by other miRNAs, including miR-27a, miR-142-5p, miR-153, miR-144, miR-93, and miR-28, the effect of these miRNAs in AITC-mediated macrophage regulation is still not clear to date (369–371). Collectively, based on miRNA-regulation, the mechanism of naturally-derived isothiocyanates on stimulated macrophages can be a potential breakthrough in the inflammation research arena.

### Phenylpropanoid

#### Cinnamaldehyde

A conjugated aromatic aldehyde, cinnamaldehyde is isolated from the barks of *Cinnamomum cassia* Presl (253). Traditionally, this plant is often used to resolve symptoms of weakness, but recently, this bioactive compound has been used in cancer, cerebrovascular diseases, and ulcerative colitis (300, 372, 373). This action has been attributed to its anti-inflammatory properties, as well as, non-coding RNA regulatory functions.

##### CA Mechanism of Action as an Anti-Inflammatory Agent

CA mitigates inflammation symptoms by decreasing the levels of ROS, COX-2, and the proinflammatory cytokines, such as TNF-α, IL-1β, and IL-6, in addition to NLRP3 inflammasome, as well as miR-155 and miR-21 in macrophages (253) (**Table 1** and **Figures 1** and **3**). Further studies linked the suppression of IL-1β and IL-6 to miR-21 or miR-155 inhibition, revealing that these inflammatory factors are positively regulated by miR-21 or miR-155 (300). Through this suppressive activity, CA performs its protective effect in ulcerative colitis (300). Similar to the aforementioned plant-derived polyphenols, CA downregulated several proinflammatory cytokines, including IL-1β, IL-6, TNF-α, and other inflammatory factors, such as NO and PGs, as well as COX-2, iNOS and NF-κB (148). Such effects of CA have been indicated in a plethora of studies using *in vitro* and *in vivo* models of LPS-induced inflammation (147). When CA was compared with the other aforementioned natural TLR4 modulators, some differences in the underlying molecular mechanisms could be highlighted. For example, CA contains α, β-unsaturated carbonyl moieties that act as electrophilic carbon and react with the nucleophilic thiol of a cysteine, subsequently forming Michael adducts with protein targets, thus disrupting the TLR4/MD-2 heterodimer. In contrast to curcumin, CA disrupts the TLR4/MD-2 heterodimer through the formation of covalent adducts (169, 297). In comparison to SFN, CA contains a different electrophilic group, an α,β-unsaturated carbonyl moiety, instead of the isothiocyanate moiety, which also forms covalent adducts with cysteine thiols of MD-2 (288). The pharmacological properties of the abovementioned phytochemicals on TLR4 signaling have been determined by mass analysis experiments on purified receptors (298). CA inhibition of LPS-induced NF-κB and IRF3 is the main molecular mechanism underlying its anti-inflammatory function (374). CA, however, does not show a significant action when NF-κB is activated by MyD88 and IKKβ downstream effectors, which confirms its upstream activity on the TLR4/MD-2 extracellular complex (374). Moreover, similar to SFN, CA inhibits LPS-induced TLR4 receptor oligomerization and activates Nrf2 pathway (298, 299) (**Table 1** and **Figure 1**).

## Summary

In a nutshell, this review focuses on the mechanisms of different plant-derived compounds involved in regulating inflammation within different cell types, such as macrophages, cardiac myocytes, adipose tissue and epithelial cells. While these mechanisms are pleiotropic and usually target multiple sites of action in the TLR4 pathway, some of them are common between different phytochemicals, and are significant to their established anti-inflammatory effects. In this context, the mentioned phytocompounds, collectively, inhibit the expression of TLR4 receptor, and block the activation of NF-κB transcription factor, thus inhibiting the generation of the downstream pro-inflammatory cytokines, such as TNF-α, IL-6 and IL-1β, and the free radicals, such as NO and ROS. Furthermore, unlike their inhibitory effect on TLR4/NF-κB cascade, these aforementioned phytochemicals activate Nrf-2 signaling pathway, which in turn, inhibits oxidative stress. On the other hand, there are also specialized specific mechanisms that are more relevant to some phytochemicals over others. For example, genisten and CA hinder the ligand-induced oligomerization of TLR4 receptor, while SFN suppresses both ligand-induced and ligand-independent oligomerization of TLR4. Moreover, CUR, SFN and CA competitively antagonize LPS binding to MD-2 binding site, and thus disrupt the TLR4/MD-2 heterodimer. This leads to the inhibition of both MyD88 and TRIF-dependent pathways, which are involved in TLR4 cascade activation. At the epigenetic level, plant-derived compounds undergo noteworthy epigenetic modifications that fine tune their anti-inflammatory functions. In this review, the majority of the mentioned phytochemicals significantly downregulated miR-155 and miR-21, both reduce NF-κB activity and suppress inflammatory factors, such as TNF-α, IL-6 and MAPKs, attenuating the induced-inflammation. Beside the regulation of miRNA, the mentioned phytochemicals play a critical role in decreasing the expression of pro-inflammatory genes and suppressing NF- κB-dependent inflammation in different cells through inhibiting HATs (e.g., p300 HAT) and HDACs (e.g., HDAC I, II and III). Furthermore, some phytochemicals, such as CUR, API, tanshinone IIA, and SFN downregulate DNMTs expression (e.g., DNMT 1, 3a, and 3b), and repress DNA hypermethylation at the promoter region of some genes, such as Nrf2, increasing their expression. To the contrary, RES, for instance, upregulates DNMT 3a and 3b expression, and thus mediates DNA hypermethylation at inflammatory genes, and therefore attenuates inflammation. Collectively, these stated similarities and differences between phytochemicals are what substantiate, in general, their remarkable anti-inflammatory effects, and in specific, their differential therapeutic potential and efficacy against certain inflammatory diseases in different cell types. Last but not least, as noticed, TLR4/NF-κB and Nrf2 pathways are crucial mechanisms that are targeted by almost all the mentioned phytochemicals. Taken together, future research should further investigate the crosstalk between the two pathways.

## Conclusion and Future Investigations

In conclusion, an evolving group of evidence has shown how plant-bioactive compounds significantly influence both health and disease *via* epigenetic modification. Although this report covered different phytochemicals used in distinct inflammatory experimental models and focused, in particular, on their epigenetic regulatory mechanisms, few studies have translated the epigenetic-mediated actions of these plant derivatives to human models and little is understood about gene regulation mediated by natural products in health and disease (27). Thus, it will be of great benefit if future research is directed to revealing the most effective phytochemicals in attenuating inflammatory-associated dysregulations, neurodegenerative and cardiovascular diseases. To our knowledge, how epigenetic regulation targets certain genes, in specific, is still elusive. For instance, the mechanism underlying histone modifications or DNA methylation in determining the patterns of transcription of PRRs and its downstream signaling mediators throughout infection and lineage differentiation remains unclear, and requires deep mechanistic investigations (29). As such, future research on innate immune cells should focus on identifying specific epitranscriptomes. Furthermore, post-transcriptional modifications in RNA have been known to modulate different signal transductions (29). In this regard, a mechanistic study on the effect(s) of the currently reported phytochemicals on an array of inflammatory-associated miRNAs would be of great benefit to understand how these natural compounds differentially modulate these miRNAs, and how they ultimately attenuate inflammatory processes. For instance, previous reports on the regulation of miR-155 by polyphenols give a clear insight into flavonoid mechanism in alleviating inflammation (120). There are still other miRNAs, which are involved in macrophage inflammatory responses, that have not been studied yet with phytochemicals, among which are miR-467b, miR-33s, and miR-125a (120). Also, studies have not revealed the effect of phytochemicals on *histone acylation*, in specific (27). This lack of information shed the light on the inevitability of investigating the mechanisms by which phytochemicals modulate histone acylation, and whether they could mitigate inflammation. In addition to epigenetic regulations, only few studies tackled the interactions taking place between phytochemicals (27); future work should therefore address phytochemical-phytochemical interactions, and elucidate the combined effect of these interactions during inflammation, particularly at the epigenetic level. Assessing phytochemical interactions sheds light on understanding the pharmacokinetics of these compounds, because these compounds usually vary in absorption, distribution, metabolism and excretion (ADME) inside the body (375, 376). Subsequently, studying pharmacokinetics variations between these phytochemicals is inevitable. This could unveil natural compounds with similar absorption rates and distribution sites to be further explored in combinations, and enable examining any potentiation of effect resulting from such combinations. Another consideration is the metabolic activity of these phytochemicals; whether the metabolites of parent phytochemicals possess a therapeutic activity or not. For example, in spite of emodin’s poor distribution to heart cells, its cardioprotective effect has been reported (221, 375). This observation has been explained in terms of metabolomics, in which blood-circulating metabolites of parent compounds exert, an efficacious activity that is observed in tissues having undetectable concentrations of the parent compounds (27). Nonetheless, most phytochemical metabolites are usually non-bioavailable (376, 377). A fact that raises the necessity to delve deeper into the area of phytochemical-related metabolomics. The poor bioavailability of several phytochemicals, especially polyphenols, is another issue to be tackled in future research. Increasing the dose of these phytochemicals is not always safe; it has to be mentioned that they, like other treatments, may exhibit toxic side effects at higher concentrations (378–380). This, therefore, brings up questions as to the phytochemical-associated toxidrome at high concentrations, and should engender explorative efforts to search for new technologies that enhance bioavailability at lower doses, such as solubilizers and targeted drug-delivery systems (381). Multiple reports suggest that co-administering plant-bioactive compounds with other pharmaceutical treatments resulted in differential gene expression, which might imply that their actions are epigenetically-related (27, 382). For example, when curcumin is co-administered with reinstate (SAHA; an FDA-approved HDAC inhibitor), an enhanced effect has been observed in ameliorating antibody-dependent neurodegeneration than that of single treatments (382). Another report showed that resveratrol co-treatment with metformin (anti-diabetic drug) successfully ameliorated inflammation, and other metabolic dysregulations in diabetic mice (383, 384). Therefore, there is a need to understand the exact epigenetic mechanism(s) that cause such synergism, and further explore approved, commercially available medications in conjunction with natural compounds.

## Author Contributions

HS and AA designed the outline of the review. MY and AA edited and proofread the review. HS, MY, and AA contributed to the writing, proof reading, and creating the table of this review. All authors contributed to the article and approved the submitted version.

## Funding

This work is supported by AUC graduate research grant to HAS and MHY and AUC internal grant [FY19-RG (1-18)], Egyptian Academy of Scientific Research and Technology Grants (JESOR-2019-5305), and (ASRT-2019-4903), a Bartlett Fund for Critical Challenges Grant and an AUC COVID-19 Pandemic Research & Innovation Initiative Grant to AA.

## Conflict of Interest

The authors declare that the research was conducted in the absence of any commercial or financial relationships that could be construed as a potential conflict of interest.
